# Environmental Impacts on Infectious Disease: A Literature View of Epidemiological Evidence

**DOI:** 10.5334/aogh.3670

**Published:** 2022-10-21

**Authors:** Peter D. Sly, Brittany Trottier, Atsuko Ikeda-Araki, Dwan Vilcins

**Affiliations:** 1 Children’s Health and Environment Program, Child Health Research Centre, The University of Queensland, Australia; 2 Superfund Research Program, National Institute of Environmental Health Sciences, USA; 3 Faculty of Health Sciences, Hokkaido University, JP; Center for Environmental and Health Sciences, Hokkaido University, Japan

**Keywords:** Oxidative stress, Covid-19, children, air pollution, cohort study, birth cohort

## Abstract

**Background:**

This article summarises a session from the recent Pacific Basin Consortium for Environment and Health Focus meeting on Environmental Impacts on Infectious Disease.

**Objective:**

To provide an overview of the literature underpinning the presentations from this session.

**Methods:**

References used in developing the presentations were obtained from the presenters. Additional references were obtained from PubMed using key words from the presentations.

**Findings and Conclusions:**

1. The Hokkaido longitudinal children’s study has found that exposure to chemicals in early life, such as persistent organic pollutants and per/polyfluorinated compounds, is associated with a range of immunological outcomes such as decreased cord blood IgE, otitis media, wheeze, increased risk of infections and higher risk of food allergy.
2. Epidemiological evidence links exposure to poor air quality to increased severity and mortality of Covid-19 in many parts of the world. Most studies suggest that long-term exposure has a more marked effect than acute exposure.
3. Components of air pollution, such as a newly described combustion product known as environmentally persistent free radicals, induce oxidative stress in exposed individuals. Individuals with genetic variations predisposing them to oxidative stress are at increased risk of adverse health effects from poor air quality.

## Environmental Chemicals and the Risk of Infectious Disease in Children

Young children and the developing fetus are especially vulnerable to adverse environmental exposures [1,2]. Pollution and environmental chemicals are recognized as causing substantial mortality [3] and adding substantially to the burden of disease [4]. While traditional pollutants, such as ambient and indoor air pollution, persistent organic pollutants, heavy metals, and pesticides are well recognized as contributing to ill health [4], there is an increasing recognition that a wider view of all environmental contributors to adverse health outcomes is required. The concept that individual toxicants rarely act alone in increasing disease risk but that all exposures must be considered is gaining traction. The totality of exposures is often referred to as the “exposome.” [5,6] The exposome includes exposure to xenobiotic toxicants, endogenous stressors resulting from exogenous exposures, toxic metabolites of exposures, dietary constituents, and psychological and physical stressors, as well as their biological responses.

Since the 1950s the world has experienced an “explosion” of synthetic chemicals introduced into our environment. While many of these have made lives easier, the safety of the vast majority of these chemicals was not tested prior to their introduction. The toxicity of many chemicals and their adverse effects on human health may only become apparent after years of use. The use of some chemicals has been banned, for example some persistent organic pollutants have been banned under the Stockholm convention [http://www.pops.int/], however many others continue to be used and untested alternatives to banned substances are frequently used. Two classes of chemical attracting recent attention are the per- and polyfluoroalkylated substances (PFAS) and the short half-life plastics additives, plasticizers, bisphenols and phthalates.

PFAS are ubiquitous in modern environments and commonly reported in human biomonitoring studies [7]. They are used to provide non-stick, waterproof and greaseproof surfaces on consumer products and have been widely used in fire-fighting foams [7]. Human exposure primarily occurs via contaminated drinking water, although exposure can also occur through contaminated dust, air, and foods [8]. In some countries, including the USA and Australia, large populations have been exposed to PFAS-contaminated drinking water, yet direct evidence of adverse health effects is scarce and controversial. Epidemiological studies have reported associations between PFAS exposures and various adverse health effects, including altered immune and thyroid function, liver disease, lipid and insulin dysregulation, kidney disease, adverse reproductive and developmental outcomes, and cancer [9]. Exposures during pregnancy have been associated with pregnancy complications, including hypertension and preeclampsia, and low birth weight in the offspring [10]. Studies in children have reported associations between PFAS exposure and febrile infections [11], respiratory tract infections [12], and asthma [13]. Prenatal exposures have also been associated with impaired vaccine responsiveness in early childhood [14], atopic dermatitis and increased respiratory and urinary infections in girls [15]. Results from different studies are not always consistent [13,15,16], with some showing effects in girls but not in boys [15,17].

Bisphenols and phthalates have endocrine-disrupting properties that adversely impact human health [18,19,20]. While these compounds have short half-lives the ubiquitous nature of their presence in modern environments mean that essentially all humans have blood levels of these compounds, and they can be measured in urine [21]. Ingested bisphenols are rapidly metabolised in the liver to an inactive conjugated form and many studies do not distinguish between free (active) bisphenols and total bisphenols. However, active free bisphenols can routinely be detected in human biomonitoring studies [22]. Free bisphenols also readily cross the placenta, influence placental gene expression, and can be measured in cord blood [23,24,25,26]. Early literature concentrated on bisphenol A but other bisphenols, including bisphenol S, bisphenol F, and bisphenol AF have attracted recent attention and have the same endocrine disrupting properties as bisphenol A [25]. Similarly, both low and high molecular weight phthalates cross the placenta, can be measured in cord blood, and have adverse effects on the developing fetus and children [19,27,28,29,30,31]. Longitudinal human birth cohort studies have contributed much to our knowledge of the adverse consequences of environmental exposures during fetal development and in early postnatal life.

This paper will discuss three important, but different, linkages between environmental exposures and immune functioning. Firstly, we will present important data from the Hokkaido Study on Environment and Children’s Health showing that chemical exposure in early life is associated with increased risk of infection and immunologically driven health outcomes. Next, we will explore the evidence for air pollution exposure and risk of Covid-19. Lastly, we will discuss how air pollution can induce oxidative stress, a process driven by the immune system, and how individuals with a genetic predisposition to oxidative stress may be most at risk of the harmful effects of air pollution.

## The Hokkaido Study on Environment and Children’s Health (The Hokkaido Study)

The Hokkaido study includes two birth cohorts, an initial cohort consisting of 514 pregnant women, recruited from one hospital in Sapporo between 2002 and 2005 at second to third trimester, and a second cohort, consisting of 20 926 pregnant women at gestational age <13 weeks, recruited from 37 hospitals in Hokkaido between 2003 and 2012 [32]. The objectives of the study were:

1. To examine the effects of perinatal environmental factors on birth outcomes, including congenital anomalies and growth retardation.
2. To evaluate the prevalence of allergic diseases, developmental, and neurobehavioral disorders.
3. To identify a high-risk group classified by genetic susceptibility and investigate trans-generational epigenetic effects of environmental chemicals.
4. To provide scientific evidence for health policies based on human epidemiological data.

The study focused on persistent organic pollutants, in other words, environmental chemicals with long half-lives, such as dioxins and polychlorinated bisphenols, organochlorine pesticides, such as DDT, PFAS and endocrine-disrupting chemicals with short half-lives but ubiquitous exposure, such as phthalate esters, bisphenols and organophosphate flame retardants [33]. Major findings from the study related to the immune functions to date include:

- Description of the cohort exposure levels of dioxins, polychlorinated bisphenols, PFAS, bisphenols and phthalates, and methyl mercury [34].
- Temporal trends of PFAS from 2003 to 2011 [35].
- Maternal to child transfer of PFAS [36].
- Reduction of cord blood IgE levels and increase in wheeze in boys to age 3 and related to higher dioxin levels in maternal blood [37].
- Higher dioxin levels in maternal blood increase risk of otitis media in boys at 18 months of age [38].
- Prenatal dioxin exposure increases risk of wheeze in both boys and girls at age 7 years [37].
- Prenatal exposure to PFAS increases risk of pneumonia in both boys and girls to age 7 years [39].
- Exposure to phthalates in house dust is associated with allergies in school-aged children [40].

Children in the Hokkaido study are currently adolescents and follow up is planned to continue into adult life. We look forward to more important findings from this study.

## Epidemiological evidence of air pollution increasing Covid-19

Since the onset of the Covid-19 pandemic there has been an explosion in publications dealing with many aspects of the Sars – Cov2 virus, clinical features of Covid-19, and epidemiological studies addressing factors increasing susceptibility to and severity of Covid-19. Knowledge in many areas remains limited, including why children were relatively spared, an unusual situation with respiratory viruses [41]. Following initial observations between poorer air quality and increased Covid-19 mortality in the USA [42], the issue of whether air pollution increases susceptibility to, and severity of Covid-19 has attracted much attention. Indeed, a PubMed search conducted on September 8^th^ 2021 using the terms “Covid-19” and “air pollution” showed that 554 articles had been published in 2020 and 690 published to date in 2021. Of these 80 in 2020 and 107 in 2021 claimed to be systematic reviews.

An association between Covid-19 and air pollution is certainly supported by the literature but is not simple. For example, some of the most polluted mega-cities in India, Bangladesh, and Africa were not initially epicentres of Covid-19. Examination of standardised mortality rates from Covid-19 for 10 age groups showed a correlation with particulate matter with a mass median aerodynamic diameter (PM_10_) [β 0.147 (95% CI 0.059–0.234), p = 0.001] across 36 Italian provinces [43]. Furthermore, measures of long-term air quality (2016-2019) across 71 Italian provinces showed associations between case numbers of Covid-19 and annual average NO_2_ (R^2^ = 0.247, p < 0.01), PM_2.5_ (R^2^ = 0.340, p < 0.01), PM_10_ (R^2^ = 0.267, p < 0.01) exposure [44].

Travaglio et al. [45] examined associations between air pollution in the UK, using either 2018 data or 5-year average data, and Covid-19 cases and mortality. Using population data, the main contributor of Covid-19 was NO_2_, with a 1µg/m^3^ increase in NO_2_ in 2018 associated with a 3.3% increase in cases and 3.1% increase in deaths. Restricting the study population to participants in the UK-Biobank, where data were available at an individual level, showed that for every unit increase in PM_2.5_ and PM_10_, the number of Covid-19 cases increased by 1.7% and 11.7%, respectively [45]. In general, the associations between air pollutants and Covid-19 cases and mortality were similar when 5-year average exposures were used instead of 2018 data. This raises the question of whether long-term air pollution exposure is more strongly associated with Covid-19 cases and mortality than short-term exposure. This was partly addressed in a Chinese study where the association between air pollution exposure and daily Covid-19 case rates were examined with lags between 0-7 days, 0-14 days, and 0-21 days [46]. For PM_2.5_, PM_10_, CO, and NO_2_ the associations were stronger with longer lags between exposure and cases [46]. These data are consistent with a study from New York City that showed no associations between PM_2.5_ and Covid deaths with a lag of 0-1 day [47]. In contrast, there was a significant association between moving average O_3_ (ppb) exposure and new Covid-19 cases. A one unit increase in moving average O_3_ was associated with as 10.5% increase in new cases [47]. One short-term exposure associated with increased Covid-19 cases is exposure to wildfire smoke. Kiser et al. [48] studied case rates of Covid-19 in Reno, Nevada, associated with a major wildfire. They reported that a 10µg/m^3^ increase in wildfire PM_2.5_ was associated with a 6.3% relative increase in SARS-Cov2 positive test rate. A review of 26 articles from many countries showed associations between short term air pollution exposure and increased cases of Covid-19, suggesting an increase in viral transmission [49]. In the same review long-term air pollution exposure was associated with increases severity and mortality [49]. One possible mechanism for this association could be alterations in the airway epithelium that make it respond to SARS-Cov2 infection with a more pro-inflammatory response. Evidence is emerging that both particulate matter and covid-19 induce angiotensin II-dependent proinflammatory cytokine production. The lung contains type II alveolar cells that express angiotensin-converting enzyme 2 receptors (ACE2). SARS-CoV-2 binds to these receptors, and through the renin-angiotensin axis induces inflammation. Particulate matter has been shown in preclinical studies to upregulate the same pathway, and lead to production of the same cytokines see in the covid-19 induced cytokine storm, thus there is the potential for both particulate matter and SARS-CoV-2 to be triggering the same pro-inflammatory targets [50].

## Oxidative stress as a mediator of the effect of air pollution on respiratory disease

The Lancet Commission report on Pollution and Health highlighted the major contribution pollution makes to global mortality and morbidity [3]. Air pollution is a major contributor to childhood deaths from pneumonia and adult deaths from chronic obstructive pulmonary disease. Evidence shows that long-term exposure to PM_10_, SO_2_ and NO_2_ is associated with an increased risk of tuberculosis, potentially through local damage to the lungs which makes them more susceptible to *Mycobacterium tuberculosis* infection [51]. The physical properties of air pollution reduce ultraviolet-B intensity at ground level, which has been linked to lower vitamin D synthesis [52]. Adequate vitamin D is required for healthy immune function, and lower levels of vitamin D have been associated with several infectious diseases including tuberculosis [53]. Air pollution contributes to poor respiratory health in a number of ways, with periods of particular developmental susceptibility being during fetal development and in early postnatal life [2]. Exposures occurring during fetal development (maternal exposures) result in impaired fetal growth and impaired lung growth, both of which result in low lung function at birth [54,55,56,57]. Exposures in early life result in increased respiratory infections, reduced lung function growth and increase life-long risk of acute and chronic respiratory disease [1,2,57,58,59,60].

While the epidemiological evidence linking air pollution exposure to poor respiratory health, including increased respiratory infections, is strong, evidence for the precise mechanisms involved is less developed. Children exposed to high levels of traffic related air pollution show biomarkers of oxidative damage in exhaled breath [61], and those with reduced or null function variations in genes related to antioxidant defence show increased adverse health effects when exposed to air pollution [62,63]. Taken together, these data suggest that the adverse effect of air pollution on respiratory health are mediated, in part, via oxidant damage in the airways.

Oxygen is an oxidant gas and the lungs have developed elaborate mechanisms to defend against oxidant stimuli. In the lungs epithelial lining fluid-containing mucus and high levels of the reduced form of glutathione provides the first line of antioxidant defence. This is augmented by enzymes that detoxify xenobiotics and dietary antioxidants, such as vitamin C. Oxidant exposures result in the production of reactive oxygen species (ROS) in the lungs. ROS are generated by neutrophils and macrophages as part of the body defence against invading microorganisms. These “intrinsic” ROS can contribute to overwhelming antioxidant defences and contribute to oxidant damage. The term “oxidative stress” is used to describe the situation where the body’s antioxidant defences are overwhelmed by ROS, resulting in tissue damage [64].

Air pollution is a complex mixture of solid, gaseous, and chemical components. Particles generally have a carbonaceous core, to which chemicals and oxidant radicals can be bound. These may include transition metals, oxidant chemicals, persistent organic pollutants, and other toxicants. The composition of particulate matter is influenced by what is burnt and how efficient the combustion process was. A relatively newly discovered oxidant product of incomplete combustion are environmentally persistent free radicals [65,66]. These are the subject of another publication in this series and will not be discussed further here. PM entering the lungs can induce direct epithelial damage, reducing the effectiveness of the epithelial barrier, reduce mitochondrial function via inducing oxidative stress, and directly stimulate inflammatory pathways. O_3_ and NO_2_ are more likely to exert their effects via stimulation of inflammatory pathways, with resultant ROS generation and induction of oxidative stress [67].

Oxidative damage to the respiratory epithelium is a likely mechanism linking air pollution exposure to increased risk of respiratory infection [68]. Impaired barrier function would allow greater access for invading microbes to the epithelial cells and sub-epithelial layers. Oxidant damage to epithelial cells may reduce the production of anti-microbial peptides, reducing anti-viral and anti-bacterial defences. Induced mitochondrial dysfunction results in reduced ATP production and increased cell death, potentially allowing greater microbial spread from cell to cell. Induction of pro-inflammatory pathways further increases ROS generation and cell dysfunction. One additional potential mechanism by which air pollution can increase risk of respiratory infections is via interfering with anti-microbial activity of Vitamin D [69]. This activity comes, in part, via upregulating transcription of the cathelicidin gene to produce the cathelicidin peptide. This is cleaved to form a cationic peptide, known as LL37, which binds to microbes, punching holes in susceptible bacteria or destroying the envelope of enveloped viruses, such as Sars-Cov2. Fine particles in air pollution appear to inactivate LL37, possibly by changing it from a cationic to a neutral peptide, removing its anti-microbial activity, and facilitating viral replication.

## Conclusion

The Pacific Basin Consortium 2021 Focus Meeting allowed research teams to come together and share valuable insights on environmental health. This paper summarises a session exploring environmental impacts on infectious disease and shows the importance of a range of environmental exposures to human health. The Hokkaido Study on Environment and Children’s Health is an invaluable birth cohort in the field of children’s environmental health and, as shown here, has generated high quality evidence of the negative association between environmental chemicals and children’s immune function. Long-time follow-up is warranted to examine how effects last and later onset of diseases. The evidence of air pollution’s role in both infectious diseases generally, and COVID-19 specifically, is well developed. The role of oxidative stress as a mediator between environmental exposures and immune health is emerging as key mechanism of interest. Globally, we find ourselves in a time of unprecedented environmental change while simultaneously adapting to the on-going effects of the Covid-19 pandemic. Understanding the complex interplay between the immune system and the environment is important to protecting public health across all populations.
